# Investing in mental health and well-being: findings from the DataPrev project

**DOI:** 10.1093/heapro/dar059

**Published:** 2011-12

**Authors:** David Mcdaid, A-La Park

**Affiliations:** 1 Personal Social Services Research Unit, LSE Health and Social Care and European Observatory on Health Systems and Policies, London School of Economics and Political Science, London, UK; 2 Personal Social Services Research Unit, LSE Health and Social Care, London School of Economics and Political Science, Houghton Street, London WC2A 2AE, UK

**Keywords:** economic evaluation, mental health promotion, children, older people, workplaces

## Abstract

A systematic review was conducted to determine the extent to which an economic case has been made in high-income countries for investment in interventions to promote mental health and well-being. We focused on areas of interest to the DataPrev project: early years and parenting interventions, actions set in schools and workplaces and measures targeted at older people. Economic evaluations had to have some focus on promotion of mental health and well-being and/or primary prevention of poor mental health through health-related means. Studies preventing exacerbations in existing mental health problems were excluded, with the exception of support for parents with mental health problems, which might indirectly affect the mental health of their children. Overall 47 studies were identified. There was considerable variability in their quality, with a variety of outcome measures and different perspectives: societal, public purse, employer or health system used, making policy comparisons difficult. Caution must therefore be exercised in interpreting results, but the case for investment in parenting and health visitor-related programmes appears most strong, especially when impacts beyond the health sector are taken into account. In the workplace an economic return on investment in a number of comprehensive workplace health promotion programmes and stress management projects (largely in the USA) was reported, while group-based exercise and psychosocial interventions are of potential benefit to older people. Many gaps remain; a key first step would be to make more use of the existence evidence base on effectiveness and model mid- to long-term costs and benefits of action in different contexts and settings.

## INVESTING IN MENTAL HEALTH AND WELL-BEING

### Economics, mental health and well-being

The personal, social and economic costs of poor mental health, much of which fall outside the health-care sector, have been well documented. In the European Economic Area alone, the costs of depression and anxiety disorders have been estimated at €136.3 billion (2007 prices). The majority of these costs, €99.3 billion per annum, are due to productivity losses from employment (Andlin-Sobocki *et al.*, 2005). Behavioural problems that arise in childhood and remain significant in adult life can increase costs not only to the health system, but also to criminal justice and social services, with reduced levels of employment and lower salaries when employed and having adverse impacts on personal relationships (Scott *et al.*, 2001; Fergusson *et al.*, 2005; Smith and Smith, 2010). Poor mental health is the leading or second most reason for early retirement or withdrawal from the workforce on health grounds (McDaid, 2011).

While these are serious impacts, they are in themselves insufficient to justify investment in measures to promote mental health and well-being. For this, it is important not only to identify robust evidence-informed actions, but also to look at their costs and resource consequences, within and beyond the health system. Resources are always finite, with many potential alternative uses, and careful choices have to be made on investment and priority setting. It is perhaps even more critical to highlight whether investment in the promotion of mental health and well-being might represent good value for money and help avoid future costs of poor mental health during the current austere climate when health and other public sector budgets are under substantial pressure, and when mental health promotion may not be seen as a high priority for policy makers (McDaid and Knapp, 2010).

As part of the EC funded DataPrev project, a systematic review was conducted to identify the state of the evidence base on the use of economic evidence in helping to make the case for investment in mental health and well-being in the four areas of focus to the project: early years and parenting interventions, actions set in schools and workplaces and measures targeted at older people.

## METHODS

Our objective was to identify economic evaluations, i.e. studies comparing the effectiveness and costs of two or more health-focused interventions, to promote mental health and well-being and/or prevent the onset of mental health problems.

### Inclusion and exclusion criteria

Two distinct types of study were eligible for inclusion. First, economic evaluations conducted concurrently or retrospectively alongside a randomized controlled trial. An exception to this criterion was applied to workplace health promotion interventions where controlled trials are rare; in this case other empirical study designs alongside an economic analysis were also eligible. Economic evaluations conducted using a modelling approach, whereby effectiveness data were collected from one or more previous controlled studies and then combined with data on costs, were also included. Economic evaluations had to be consistent with different approaches commonly applied in health economics, including cost-effectiveness, cost–benefit, cost-consequence, cost–utility and cost-offset analyses. While we cannot discuss the differences between these approaches here, the interested reader can refer to numerous guides, e.g. (Drummond *et al.*, 2005; Shemilt *et al.*, 2010).

To be eligible for inclusion studies also needed to include either a measure of positive mental health, e.g. use of the SF-36 mental health summary scale or other measures of quality of life, specific measures of well-being or alternatively quantify the prevention of psychosocial stress and/or mental disorders. We excluded studies relating to the prevention of dementia, as well as those focused on individuals with learning difficulties from our analyses. Interventions needed to have a primary objective of promoting health. This meant that we excluded some education and child care centred interventions that had subsequently been shown to have a positive impact on mental health (among other outcomes) (Barnett, 1998; Barnett and Masse, 2007).

Papers that focused on the treatment of individuals with existing mental health problems were excluded, with the exception of studies that looked at how the treatment of parents with mental health problems might promote/protect the mental health of their children, as well as those reporting proxy outcomes, such as improvements in parent–child interaction and the prevention of child abuse. Children were assumed to be between the ages of 0 and 16, while studies in respect of older people focused on people aged 65 plus.

### Search process

A search strategy designed to identify economic evaluations in bibliographic databases (Sassi *et al.*, 2002) was combined with a range of mental health promotion/mental disorder terms and a set of population/setting specific keywords and phrases. Mental health-related terms and concepts included in the search included mental health, positive mental health, mental and emotional well-being, personal satisfaction, quality of life, happiness, resilience, energy and vitality. Health promotion and prevention-related keywords and phrases were also combined with terms related to poor mental health, including psychological stress, post-natal/post-partum depression, conduct disorder and child behavioural disorders.

We searched PubMed, PsycINFO, EMBASE, CINAHL, PAIS, Criminal Justice Abstracts, Web of Science, Scopus, EconLit and the National Health Service (NHS) Economic Evaluation Database at the University of York. Only results that reported abstracts (or chapter summaries) in English were included; geographical coverage was limited to the European Economic Area, plus EU Candidate Countries, Switzerland and other Organisation for Economic Co-operation and Development (OECD) members. Our review covered the period from January 1990 to December 2010. The electronic search was complemented by a limited search for key terms in Google Scholar, the general Google search engine and scrutiny of relevant websites, e.g. think tanks, universities, government departments and agencies. We also undertook a handsearch of a small number of journals and examined the reference lists of included studies, as well as citations of papers that met our inclusion criteria.

In addition, we also looked for any economic analyses of mental health promoting interventions previously shown in companion systematic reviews on effectiveness conducted as part of the DataPrev study to be effective in promoting mental health and well-being. Where these reviews identified evidence of the impact of an intervention on mental health and well-being, any studies that looked at the economic case for investment in those interventions, even if focused on non-health benefits, such as improved educational attainment, reduced crime and violence, were then eligible for inclusion.

**Fig. 1:**
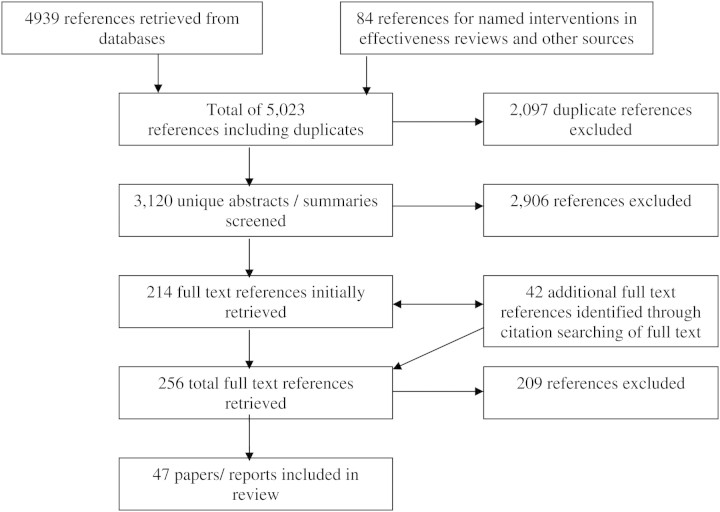
Search flow chart.

References were initially screened independently by two reviewers (D.M. and A.P.) on the basis of their abstracts/summaries to determine whether they met study inclusion criteria. In the case of disagreement the two reviewers discussed the paper and came to a final decision on inclusion/exclusion, erring on the side of inclusion where no easy agreement could be reached. The full text of all references appearing to meet initial inclusion criteria was then retrieved and a final assessment made. Ultimately included studies were coded and stored in an Endnote database. An assessment of the quality of studies was also made, making use of two published economic evaluation checklists (Drummond and Jefferson, 1996; Evers *et al.*, 2005). Overall this process meant that >3000 references were assessed (see Figure 1).

## RESULTS

### Parenting, early years and school-based interventions

There has been a considerable body of research into the effectiveness of interventions to promote/protect the mental health and well-being of children and their parents, both within and external to school settings (Adi *et al*., 2007a, b; Dretzke *et al.*, 2009); there is also a small but growing number of studies looking at the economic case for taking action, albeit largely set in either a USA or UK context. We also identified one study protocol for an economic evaluation of an internet-based group intervention to prevent mental health problems in Dutch children whose parents have mental health or substance abuse problems (Woolderink *et al.*, 2010). Overall the results are mixed, as the summary of findings from 26 papers and reports in Tables 1 and 2 indicate.

**Table 1:** Economic analyses alongside empirical studies of parenting, early years and school-based interventions promoting mental health and well-being

Bibliographic information	Intervention (I) and comparator (C)	Target population and duration of economic analysis	Study design	Cost results	Mental health-related effectiveness results	Perspective/price year	Synthesis of costs and effectiveness data
(Cunningham *et al.*, 1995), Canada	I: Large group community-based parenting programmes	Parents of 150 pre-school/kindergarten children at high risk of developing conduct disorders	RCT	Community-based groups were reported to be more than three times as much as clinic/individual parenting sessions	Community group had a significantly greater number of solutions to problems than control groups (*p*< 0.05) Significantly better in reducing behavioural problems at home compared with the clinic group (*p*< 0.05). Community group reported greater improvement than the clinic group, but significantly better parental sense of competence in the clinic control group (*p*< 0.05)	Health sector and travel costs	No synthesis of costs and benefits. Community-based group reported have better outcomes than clinic-based programmes and to be six times more cost-effective because of higher number of people reached by group sessions
	C: Clinic-based individual parenting programmes or 6 months waiting list	6 months	CCA			CAD. Price year not stated	
(Edwards *et al.*, 2007), Wales	I: The Webster-Stratton Incredible Years group parenting programme	Parents of 116 children aged 36–59 months at risk of developing conduct disorders	Pragmatic	The mean cost per child attending the parenting group: £934 for 8 children and £1289 for 12 children containing initial costs and materials for training group leaders.	Risk of conduct disorder linked with child behaviour. Significant improvement in mean intensity scores for child behaviour on Eyeberg scale in the intervention group of 27 points compared with no change in the control group (*p*< 0.0001)	A multiagency public sector perspective: health, special educational and social services	Incremental cost per five point improvement on the Eyeberg intensity scale would be £73. Given a ceiling ratio of £100 per point change 83.9% likelihood of being cost-effective
	C: 6 months waiting list	6 months	RCT	Incremental costs of all health, social and special education services were £1992.29 compared with £49.14 in the control group		2004 GBP	Estimated to cost £5486 to bring child with highest intensity score below clinical cut-off for risk of developing conduct disorders
			CEA				
(Foster, 2010), USA	I: Fast Track intervention: multi-year, multi-component prevention programme targeting antisocial behaviour and violence. Includes curriculum based on the PATHS programme which focuses on social and emotional learning. Includes parent training, home visiting, academic tutoring, social skills training	891 children identified at first year of entry to school system and provided intervention services over a 10-year period	RCT	Intervention cost $58 000 per child. Average health service costs (excluding programme costs) per child were $2450 in the intervention group	Focus on broad range of long-term outcomes that are associated with onset of conduct disorder in childhood: delinquency, school failure and use of school services, risk of substance abuse. No significant intervention effects were found	Public purse	No ratio reported the author states that ‘the most intensive psychosocial intervention ever fielded did not produce meaningful and consistent effects on costly outcomes. The lack of effects through high school suggests that the intervention will not become cost-effective as participants progress through adulthood’ (Foster, 2010)
	C: No intervention		CEA			2004. USD	
(Foster *et al.*, 2008), USA	Population wide implementation of multi-level Triple P intervention. (see Mihalopoulos *et al.*, 2007)	Parents and children in nine counties in South Carolina	Ongoing RCT in South Carolina	The costs for universal media and communication components: less than $0.75 per child in population	Outcomes of intervention are not reported here. Instead a threshold analysis conducted to identify costs that could be avoided if programme effective. Thresholds in line with those reported in previous studies	Programme costs plus costs to participants of various events	Estimated that the cost of implementing Triple P could be recovered in 1 year by a 10% reduction in child abuse and neglect
			COA	Total costs of providing interventions from levels 2–5 $2, 183, 812 or cost per family of $22 or $11.74 per child		USD. Price year not stated	
(Foster *et al.*, 2007), USA	I: Incredible Years Programme with three components: a child-based training programme (CT), a parent-based training programme (PT) and a teacher-based training programme (TT).	459 children aged 3–8 not receiving mental health treatments and their parents	Six RCTs	The total cost per child was $1164 with CT, $1579 with PT, $2713 with CT and PT, $1868 with PT and TT, $1454 with CT and TT and $3003 with CT, PT and TT	Parent–child interaction measured using Dyadic Parent–Child Interactive Coding System–Revised (DPICS-R; observer reported). Preschool behaviour measured using Behar Preschool Behavior Questionnaire (PBQ; teacher reported) used	Intervention costs to health and education system, including travel and refreshments and childcare costs	If payers have willingness to pay of $3000 per unit of improved behaviour on PBQ then PT and TT treatment are most cost-effective, while for values lower than $3000 no treatment was the preferred strategy
	Each component focused on improving children's behaviour through the promotion of socially appropriate interaction skills.	Data taken from six clinical trials	CEA		Parent–child interaction improved significantly for all intervention groups, except CT only. Preschool behaviour improved significantly all treated groups except for the CT, PT and TT group	2003 USD	If parent–child interaction improvement then if willingness to pay of $2500 per unit of effectiveness, the CT, PT and TT option was the most cost-effective in almost 70% of cases
	C: Comparisons were made between different combinations of the three components plus no intervention	To end of delivery of Incredible Years programme					
(Foster and Jones, 2006, 2007), USA	I: Fast Track intervention: multi-year, multi-component prevention programme targeting antisocial behaviour and violence. Includes curriculum-based on the PATHS programme which focuses on social and emotional learning. Includes parent training, home visiting, academic tutoring, social skills training	891 children identified at first year of entry to school system and provided intervention services over a 10-year period	RCT	The average cost $58 283 per participant	Diagnosis of conduct disorder using the Diagnostic Interview Schedule for Children Self Report of Delinquency instrument for violence	Public purse	Cost per case of conduct disorder averted: $3 481 433 for all population; $752 103 for high-risk individuals
			CEA		Effectiveness outcomes are not explicitly reported in paper—only the incremental cost-effectiveness ratios	2004, USD	Cost per act of inter-personal violence prevented $736 010
	C: No intervention		10 years				Intervention not considered cost effective for lower risk groups Would be cost-effective for highest risk groups if societal willingness to pay above $750 000
(Hiscock *et al.*, 2007), Australia	I: Advice and education from maternal and child health nurses to improve infant sleep and maternal well-being.	328 mothers reporting infant sleep problems at 7 months	Cluster RCT	The mean cost for intervention: £96.93 versus control family: £116.79. (non-significant difference)	Significant reduction in reported infant sleep problems at 10 months for the intervention group : 56 versus 68% (*p* = 0.04) and at 12 months 39 versus 55% (*p*= 0.007). Significant mean difference in risk of post-natal depression for the intervention group—1.4 on Edinburgh Post Natal Depression Scale (*p*< 0.007); significantly improved mental health scores on SF-12 for intervention—mean difference 3.9 *p*< 0.001	Health-care perspective	Ratio not reported as intervention dominant: lower costs, higher benefits
		5 months	CCA			(MCH sleep consultations, other health-care services and interventions costs)	
	C: Usual consultations at Maternal and Child Health Centres					GBP. Price year not stated	
(McIntosh *et al.*, 2009) and (Barlow *et al.*, 2007), England	I: An intensive home visiting programme	131 vulnerable families at risk of abuse and neglect	Multicentre RCT	Health service only: intervention £5685 versus control £3324	Statistically significant improvement in maternal sensitivity and infant co-operativeness components of the CARE Index outcome measure. Maternal sensitivity 9.27 in the intervention group versus 8.20 in the control group (*p*= 0.04)	Health and societal perspectives	No ratio assessing cost-effectiveness per unit improvement in maternal sensitivity or infant co-operativeness
	C: Care as usual	18 months	CCA	Societal costs: intervention £7120 versus £3874 for control	Infant co-operativeness 9.35 versus 7.92 in the control group (*p*= 0.02)	2004 GBP	However, cost per child identified as being at risk of neglect would be at least £55 016
					0.059 rate increase in (non-significant increase in protection of children from abuse and neglect		
(Morrell *et al.*, 2000), England	I: Post-natal support from a community midwifery support workers: practical and emotional support, to help women rest and recover after childbirth	523 new mothers aged 17 plus	RCT	At 6 months, the intervention group had significantly meant higher costs of £180. (equivalent to cots of support worker)	No evidence of significant difference in health status between groups using SF-36 or in post-natal depression using the Edinburgh Post Natal Depression Scale at 6, 6 weeks or 6 months	Health service	No ratio reported as comparator dominant with lower costs and no difference in outcomes
	C: Standard midwife care, plus up to 10 visits from support workers during first 28 days	6 weeks and 6 months	CCA	At 6 months these differences persisted with mean cost of £815 in the intervention group versus £639 in the control group		1996 GBP	
(Morrell *et al.*, 2009), England	I: Health visitor delivered psychological interventions, cognitive behavioural approach (CBA) or person-centred approach (PCA)+ SSRI	418 women at high risk of post-natal depression	Pragmatic randomized cluster trial	No significant difference in costs at 6 months between intervention and controls: £339 versus £374	At 6 months 45.6% of women in the intervention group compared with 33.9% of control found to be at risk of post-natal depression with scores >12 on the Edinburgh Post-Natal Depression Scale (*p*= 0.028)	NHS and social service perspective	No ratio and intervention dominant with similar or lower costs and better outcomes. In sensitivity analysis 90% chance of being cost-effective if threshold between £20 000–30 000 per QALY gained
	C: Health visitor usual care	6 months; analysis at 12 months of small sample only	CCA		SF-6 used to generate Quality Adjusted Life Year values. Incremental gain of 0.003 QALYs in the intervention group (0.026 versus 0.023)	2005 GBP	In a small sample at 12 months intervention also dominant
			CUA				
(Niccols, 2008), Canada	I: Eight session parent group ‘Right From the Start’ (RFTS) to enhance skills in reading infant cues and responding sensitively	76 mothers of infants	RCT	The mean costs per person per session were significantly lower for intervention: RFTS: $44.04 versus home visiting: $91.26 (*p*< 0.001)	No significant differences in outcomes on infant attachment security (measured by Attachment-Q set AQS) or maternal sensitivity (measured using Maternal Behaviour Q-score)	Health system plus parental travel costs	No incremental cost-effectiveness ratio as lower cost and better outcomes. Average cost per gain in A QS score for intervention was $430.08 compared with $1283.54. In sensitivity analysis for every $100—Return on investment three to eight times greater than for home visiting
	C: Routine health visiting	8 months	CEA			CAD. Price year not stated	
(Olds *et al.*, 1993), USA	I: Home visiting programme, social support for mother until child is age 2	400 new mothers. Emphasis on teenage, single and low-income mothers; but also other mothers	RCT	For whole population incremental programme cost $3246	Health outcomes reported in other papers, including positive effects on child mental health/risk of abuse/maternal mental health	Societal	Net costs of $1582 per mother for whole population. Net savings of $180 per mother in the low-income group
	C: Screening for developmental problems at 2 years; free transportation to regular prenatal and well-child care local clinics	48 months	COA	For low-income population incremental programme cost $3133		1980. USD	
			Societal	Economic analysis focused on long-term costs of government programmes assumed to be influenced by improved maternal and child health			
(Petrou *et al.*, 2006), England	I: Health visitor delivered counseling and support for mother–infant relationship	151 expectant mothers at high risk of post-natal depression	RCT	Mean intervention group costs per mother–infant pair were £2397 versus £2278 in the control group. Non-significant difference of £119.50	There was a non-statistically significant difference in time spent with post-natal depression (9.57 weeks in the intervention group versus 11.71 weeks in the control group)	Health and social care perspective	Incremental cost per depression free month gained of £43
	C: Routine primary care	18 months	CEA			2000; GBP	If willingness to pay of £1000 for preventing 1 month of post-natal depression, intervention 71% chance of being cost-effective (71%) with mean net benefit of £384
			CBA				
(Scott *et al.*, 2010), England	PALS study (Primary Age Learning Skills Trial)	174 children in very deprived areas of London from diverse ethnic backgrounds (76% were from minority groups)	RCT	The programme cost was £1343 per child. Total cost of the programme was £176 000	Child behaviour problems (measured through observation and Parent Account of Child Symptoms Schedule. Conduct scale of Strengths and Difficulties Questionnaire (SDQ) also completed. Parenting monitored using approach of Conduct Problems Research Programme. No significant differences in outcomes were reported with the exception that the intervention group had greater use of child centred parenting and more use of calm discipline	Study funder plus health service	No ratio provided. Authors stated programme may need to be designed to increase parent uptake and engagement to be cost-effective
	I: Basic Incredible Years Parenting Programme (12 weeks) plus 6 weeks manualized SPOKES (Supporting Parents on Kids Education in Schools) Literacy programme to help parents interact with children over books they are using l + SPOKES (6 weeks)→Primary Age Learning Skills (PALS)		CCA			GBP price year not stated	
	C: No intervention						
(Wiggins *et al.*, 2004, 2005), England	I: Supportive listening home visits by a support health visitor (SHV) or year of support from community groups (CG) providing drop in sessions, home visiting and/or telephone support	731 culturally diverse new mothers living in deprived inner city London	RCT	There were no significant differences in total costs between those in SHV, CG and control groups after 12 or 18 months although the interventions tend to be more costly: the 18 month mean costs estimated to be £3255, £3231 and £2915, respectively	Maternal depression was measured at 8 weeks and 14 months post-partum using Edinburgh post-natal depression scale (EPDS). General health questionnaire (GHQ12) used at 20 months post-partum	Public sector, voluntary groups and mothers	No ratio reported as no difference in outcomes found
	C: Standard health visitor services	12 and 18 months	CUA			2000 GBP	No net economic cost or benefit of choosing either of the two interventions or standard health visitor services

RCT, randomized controlled trial; CBA, cost–benefit analysis; CEA, cost-effectiveness analysis; CCA, cost-consequences analysis; CUA, cost–utility analysis; COA, cost-offset analysis.

**Table 2:** Economic modelling analyses of parenting, early years and school-based interventions promoting mental health and well-being

Bibliographic information	Intervention (I), comparator (C) and study population	Sources of model parameters	Type of model and timeframe	Intervention cost	Perspective/price year	Economic results
		Study population	Economic analysis			
(Aos *et al.*, 2004), USA	I: Nurse–Family Partnership for low-income women: intensive visiting by nurses during pregnancy and the first 2 years after birth to promote child's development and provide instructive parenting skills to the parents	Systematic review and meta-analysis of evaluations of trials of preventive programmes conducted since 1970. Five trials identified	Decision analytical modelling	Cost of programme over 2.5 years: $9118	Societal	Total benefits $26 298. Net benefits $17 180. Benefit to cost ratio: 2.88 to 1 including primary recipient crime avoided: $14 476; secondary programme recipient: $1961;child abuse and neglect: $5686; alcohol: $541; illicit drugs: $309
	C: Screening for developmental problems at 2 years; free transportation to regular prenatal and well-child care local clinics	Cost of programme from Olds (2002)	To age 74		2003. USD	
		Review of literature and statistics to estimate cost offsets of effective action	CBA			
		Parents and children. Low income and at-risk pregnant women bearing their first child				
(Aos *et al.*, 2004), USA programmes	I: Home visiting programmes for at-risk. Mothers and children: including instruction in child development and health, referrals for service or social and emotional support	Systematic review and meta-analysis of evaluations of trials of preventive programmes conducted since 1970	Decision analytical modelling;	Costs: $4892	Societal	Benefits: $10 969. Net benefits: $6077 including child abuse and neglect avoided: $1126; alcohol: $107; illicit drugs (disordered use): $61
	C: Usual care	13 trials identified	To age 74	Synthesis of cost from a number of different home visiting projects	2003. USD	
		Cost of programme from multiple papers in literature review	CBA			
		Review of literature and statistics to estimate cost offsets of effective action				
		Mothers considered to be at risk for parenting problems in terms of age, marital status and education, low income, mothers testing positive for drugs at the child's birth				
(Aos *et al.*, 2004), USA	I: Comprehensive school programme to reduce risk and bolster protective factors to prevent problem behaviours. Includes classroom, school and family involvement elements. Known as Caring School Community (CSC) or Child Development Project	Systematic review of evaluations of trials of preventive programmes conducted since 1970. One trial identified. Battistich *et al.* (1996)	Decision analytical modelling	Cost of programme per participant $16 over 2 years (based on personal communication with programme co-ordinator)	Societal	Costs: $16; benefits: $448
	C: No intervention	Programme costs from personal communication with programme co-ordinator	To age 74		2003. USD	Benefit to cost ratio: 28.42 to 1
			CBA			No mental health impacts included in benefits which covers drugs and alcohol only
(Aos *et al.*, 2004), USA	I: ‘Behavioural Vaccine’ to encourage good behaviour at school. A ‘Good Behaviour Game’ is regularly played with prizes given to winning teams (who have better behaviour)	Systematic review of evaluations of trials of preventive programmes conducted since 1970. One trial identified. Kellam and Anthony (1998) focusing solely on tobacco	Decision analytical modelling	Costs: $8	Societal	Benefit to cost ratio: 25.92 to 1. But benefits only look at tobacco consumption avoided
	C: No intervention	Review of literature and statistics to estimate cost offsets of effective action	To age 74	Benefits: $204	2003 USD	
		Hypothetical children in first 2 years of school	CBA			
(Aos *et al.*, 2004), USA	I: Seattle Social Development project: to train teachers to promote students ‘bonding to the school, to affect attitudes to school, behaviour in school, plus parent training'. Delivered for 6 years	Systematic review of evaluations of trials of preventive programmes conducted since 1970. One trial identified. Hawkins *et al.*, (1999, 2005)	Decision analytical modelling	Costs: $4590	Societal	Benefits: $14 426
	C: No intervention	604 children from age 6 in high-crime urban areas in non-randomized controlled empirical study	To age 74		2003 USD	Benefit to cost ratio: 3.14 to 1.
			CBA			Benefits: crime: $3957; high school graduation: n: $10 320; K-12 grade repetition: $150
(Embry, 2002), USA	I: ‘Behavioural Vaccine’ to encourage good behaviour at school. A ‘Good Behaviour Game’ is regularly played with prizes given to winning teams (who have better behaviour)	*Ad hoc* review of literature on effectiveness. Additional data on budgetary impact from unrelated work in Wyoming	Decision analytical modelling	Implementation cost: $200 per child per year versus medication costs: $70 per child per month for children with behavioural problems	Health and education	If GBG cost $200 per child per year to implement for 5000 5 and 6 year olds, there would be potential costs averted of $15–20 million from a 5% reduction in special education placement, 2% reduction in involvement with corrections and 4% reduction in lifetime prevalence of tobacco use
	C: No intervention	Hypothetical 5000 5- and 6-year-old children at school in Wyoming	Lifetime		USD. Price year not stated	
			COA			
(Hummel *et al.*, 2009), England	I: Whole school intervention to promote emotional and social well-being in secondary schools. Involves classroom intervention and peer mediation	Effectiveness data taken from paper identified through systematic review (Evers *et al.*, 2007)	Decision analytical modelling	The estimated net total cost for a school with 600 pupils aged 11–16 is £9300 per year, or £15.50 per pupil per year	Education sector;	If intervention can reduce victimization by 15%, then cost per QALY gained of £9600. At a threshold of £20 000 it is 82% probable that the intervention is cost-effective, and at a threshold of £30 000, 92% probable
	C: No intervention	Hypothetical 600 school children aged 11–16	Lifetime	Classroom intervention: £7300; peer mediation: £3900; teacher time saved £1900	GBP. Price year not stated	
			CUA			
			COA			
(Karoly *et al.*, 1998, 2005), USA, Outcome data from Olds *et al.*, (1997)	I: Home visiting programme; social support for mother until child is age 2	Data for high- and low-risk women taken from original outcome data of a Nurse–Family Partnership evaluation by Olds *et al.* (1997)	Decision analytical modelling	Cost of programme: $7271	Societal	Benefit to cost ratio:
	C: No intervention	Costing analysis builds on previous costings reported by Olds *et al.* (1993)	Lifetime	Monetary benefits to society include costs averted to public purse (including health and crime), additional income of mothers, reduction in victim costs of crime	2003 USD	High risk: 5.7 to 1 ($41 419: 7271)
		400 new mothers. Emphasis on teenage, single- and low-income mothers; but also other mothers	CBA			Low risk: 1.26 to 1 ($9151: $7271)
(McCabe, 2008), England	I: Universally delivered school-based PATH programme with three sessions per week of teacher led intervention; 10 weeks parent training	Systematic review of literature to identify (limited) effectiveness data	Decision analytical modelling	Cost per child per annum £125	Education sector	If positive impacts on emotional functioning only is £10 594 per QALY gained. Probability that cost per QALY is <£30 000 per QALY is 65%
	C: No intervention	Hypothetical cohort of children aged 7	3 years		2008 GBP	If the intervention impacts upon school performance (cognitive functioning) and emotional functioning, then £5500 per QALY. Prob QALY being <£30 000 is 66%
			CUA			
(Mihalopoulos *et al.*, 2007) and Turner *et al.* (2004), Australia	Triple P-Positive Parenting Programme, compared with no intervention	Systematic review that identified five RCTs on Triple P	Decision analytical modelling	The annual cost of implementing	‘Government as third part funder’ within health sector and criminal justice and education	Triple P has better outcomes and costs are outweighed by conduct disorder averted as long as prevalence of conduct disorder at least 7%
	Level 1: media and communication strategy targeting all parents	Children aged 2–12 years at risk of developing conduct disorders	To age 28	Triple P in Queensland to 572 701 children aged 2–12 years would be: AUD 19.7 million	2003 AUD	To pay for itself 1.5% of cases of conduct disorder would have to be averted per annum
	Level 2: 1–2 session intervention;		CEA	The cost for each level of intervention would be		
	Level 3: more intensive but brief 4-session primary care intervention;		COA	Level 1: AUD 240 000		
	Level 4: 8–10 session active skills training programme;			Level 2: AUD 5.8 million		
	Level 5 targets parenting, partner skills, emotion coping skills and attribution retraining for the highest risk families			Level 3: AUD 5.7 million		
				Level 4: AUD 4 million		
				Level 5: AUD 3.6		
				The average cost per child: AUD 34		
				The cost of implementing Triple P to one cohort of 2 year olds would be AUD 9.6 million. The average cost per child in the cohort would be AUD 51		

RCT, randomized controlled trial; CBA, cost–benefit analysis; CEA, cost-effectiveness analysis; CCA, cost-consequences analysis; CUA, cost–utility analysis; COA, cost-offset analysis.

#### Empirical studies

Table 1 includes several studies looking at the impact of health visitors, including the well-cited Nurse Family Partnership programme developed in New York in the 1980s (Olds *et al.*, 1993). Focusing on new mothers, but with a special emphasis on teenage, single- and low-income mothers, the study followed 400 mothers and their children over a 15-year period. Looking at a broad range of outcomes going beyond positive maternal and child mental health outcomes, an initial analysis reported net costs per woman of $1582 (1980 prices) over the first 4 years for the whole population, but net savings of $180 per high-risk woman (Olds *et al.*, 1993).

Home visiting programmes have also been examined in England; some focused directly on child mental well-being, others on avoiding post-natal depression, a risk factor for poor child mental health (Murray, 2009). A controlled trial of an intensive home visiting programme and social support programme for vulnerable families where children could be at risk of abuse or neglect reported a cost per unit improvement in maternal sensitivity and infant cooperativeness of £3246 (2004 prices) (Barlow *et al.*, 2007; McIntosh *et al.*, 2009). The challenge with such a finding, however, is judging whether this well-being improvement represents value for money, as it uses a clinical outcome measure which cannot be compared with other uses of resources within the health-care system. Both cost–utility analyses where outcomes are measured in a common metric, such as the Quality Adjusted Life Year (QALY) where a maximum cost per QALY deemed to be cost effective can be determined in different contexts, or cost–benefit analyses where both outcomes and costs are measured in monetary terms can be used to overcome this problem, although neither approach is without its own limitations (Kilian *et al.*, 2010).

In England, a randomized controlled trial of health visitor delivered psychological therapies for women at high risk of post-natal depression improved outcomes at lower costs than health visitor usual care. There was a 90% chance that the cost per QALY gained would be <£30 000; a level generally considered to be cost effective in an English context (Morrell *et al.*, 2009). Another trial of women at high risk of post-natal depression compared health visitor delivered counselling and support for mother–infant relationships to routine primary care, finding that if society was willing to spend £1000 to prevent 1 month of post-natal depression then the intervention would have a 71% chance of being cost effective with mean net benefits of £384 (2000 prices) (Petrou *et al.*, 2006). This contrasted with an earlier study on the use of post-natal support workers to reduce the risk of post-natal depression which did not appear cost effective (Morrell *et al.*, 2000). However, the former study needs to be interpreted carefully as neither the change in costs or outcomes in the trial were significant and a comparable measure such as the QALY was not be used. Covering a longer time period and looking at additional benefits to children and mothers may have strengthened study findings.

Compared with standard health visitor care, no effectiveness or economic benefit was found in making use of supportive home visits to ethnically diverse mothers in London (Wiggins *et al.*, 2004, 2005). Home visiting was also compared with participation in a mother–child attachment group intervention in Canada. While no difference in effects was reported, costs were significantly lower in the attachment group (Niccols, 2008). We also found a recent Australian study that reported that the provision of advice and materials within a maternal and child health centre to mothers of infants with sleep problems had similar costs but better mental health outcomes for mothers and improved sleep patterns for infants compared with standard clinic consultations (Hiscock *et al.*, 2007).

As Table 1 indicates, a number of economic evaluations of parenting studies conducted alongside randomized controlled trials have been published, some set in schools, others focused on pre-school age children. In addition we identified one published study protocol for an ongoing evaluation in Wales (Simkiss *et al.*, 2010). An evaluation of the Webster-Stratton Incredible Years parenting programme in Wales, while finding the intervention to be cost-effective for all 3–5-year-old children at risk of conduct disorder, suggested that the intervention would be most cost-effective for children with the highest risk of developing conduct disorder (Edwards *et al.*, 2007). Analysis from a trial looking at 3–8-year-old children in the USA also suggests that combining the parenting component of Incredible Years with child-based training and teacher training, even though more expensive, can be more cost-effective (Foster *et al.*, 2007).

As with many health promotion interventions, benefits are only achieved if there is uptake and continued engagement with an intervention over a period of time. One Canadian study looked at community group versus clinic-based individual parenting programmes; while both approaches were effective in reducing the risk of conduct disorders the community group approach was six times more cost-effective because it reached a larger number of parents (Cunningham *et al.*, 1995). A trial of the Incredible Years Programme, combined with a manualized intervention using reading to promote interaction between disadvantaged parents and their children in London, would however only be cost-effective if uptake and engagement rates could be improved (Scott *et al.*, 2010).

The most negative studies were linked to empirical analysis of the Fast Track programme, a 10-year, multi-component prevention programme implemented in four areas in the USA and focused in part on the promotion of better mental well-being and the prevention of antisocial behaviour and violence. Although this included as one component a school curriculum approach based on PATHS (Promoting Alternative Thinking Strategies), it did not appear to be cost-effective. This may have been partly due to limitations in outcomes data in the study, but even if the intervention could be targeted solely at high-risk children it would only be cost-effective if society was willing to pay more than $750 000 (2004 prices) per case of conduct disorder averted (Foster and Jones, 2006, 2007; Foster, 2010). In all of these Fast Track studies no specific monetary valuation was placed on the maintenance of better mental health and well-being, but rather on the long-term consequences to non-health sectors, such as criminal justice.

#### Modelling studies

As Table 2 indicates, economic models have been used to estimate some of the long-term potential costs and benefits associated with parenting, early years and school-based interventions. Further economic analysis, drawing on 15-year outcome data (Olds *et al.*, 1997) suggested that the economic case for home visiting for all women was much stronger, given the impacts it had in terms of reducing abuse, violence, the need for social welfare benefits and improved employment prospects (Karoly *et al.*, 1998, 2005). Benefits outweighed costs by a factor of 5.7 to 1 for high-risk women and 1.26 to 1 for low-risk women.

As part of a wide-ranging economic analysis of early intervention programmes commissioned by the Washington State Legislature, several programmes relevant to DataPrev were modelled. It should be noted that the authors of these analyses acknowledged that a limitation of their modelling analysis was that it did not put a monetary value on the economic benefits associated with gains in social and emotional mental well-being or broad health benefits. This was due to the terms of the reference received from the Washington State Legislature, which limited the outcomes for all evaluations to crime, substance abuse, educational outcomes, teenage pregnancy, teenage suicide attempts, child abuse, neglect and domestic violence (Aos *et al.*, 2004).

Nonetheless this Washington State review included further evidence of an economic case for action. Analysis of the Nurse Family Partnership, making use of further updated cost data (Olds *et al.*, 2002) reported a benefit to cost ratio of 2.88 to 1 when modelling benefits to child school leaving age, with major benefits due to crime avoided (Aos *et al.*, 2004). Combining data from several similar home visiting programmes a benefit: cost ratio for programmes targeting high-risk mothers had a 2:1 return on investment, with net benefits per mother of $6077 (2003 prices). (Aos *et al.*, 2004).

Turning to school-based interventions, the Caring School Community scheme developed in the USA (Battistich *et al.*, 1996) and now being implemented in Europe, can be delivered at a cost of $16 per pupil over 2 years, and potentially generate a return on investment of 28:1, even when just looking only at benefits of reduced drug and alcohol problems alone (Aos *et al.*, 2004). Using data from the Seattle Social Development Project, which implemented a teacher and parent intervention including child social and emotional development for 6 years and then followed up these children from age 12 to 21 (Hawkins *et al.*, 2005), costs of $4590 (2003 prices) per child were outweighed by benefits that were three times as great. Again this analysis may be conservative, as no monetary value was placed on the significant improvements seen in mental and emotional health (Aos *et al.*, 2004).

Another school-based intervention that has been modelled is the Good Behaviour Game (GBG), an approach which seeks to instil positive behaviours in children through participation in a game, with prizes given to winning teams who behave better. Potential net cost savings of between $15 and $20 million might be achieved for a hypothetical cohort of 5- and 6-year-old children if the programme could achieve a 5% reduction in special education placements, a 2% reduction in involvement with prison services and a 4% reduction in lifetime prevalence of tobacco use (Embry, 2002). Focusing solely on the economic benefits from evidence on a reduction in tobacco use rather than on any of its mental well-being benefits (Kellam and Anthony, 1998), another analysis of the GBG reported a return of investment of 25:1 (Aos *et al.*, 2004).

As Table 2 shows several economic models have looked at the case for investing in the multi-component, manualized multi-level Triple P-Positive Parenting Programme in a number of different settings. Modelling the potential benefits of universal application of Triple P to the Queensland child population aged 2–12, the average cost per child would be AUD 34 (2003 prices). It would appear to offer very good value for money when assumed to reduce the prevalence of conduct disorder by up to 4%, generating cost-savings of AUD 6 million. The intervention would have better outcomes and costs would be outweighed by conduct disorder averted as long as the prevalence of conduct disorder was at least 7% (Turner *et al.*, 2004; Mihalopoulos *et al.*, 2007). In a USA context, an economic model predicted that the costs of Triple P could be recovered in 1 year through a modest 10% reduction in the rate of child abuse and neglect (Foster *et al.*, 2008).

In England, modelling work for NICE (National Institute for Health and Clinical Excellence) looking at the universal use of a teacher delivered PATHS programme for children combined with parent training was reported to have a 66% chance of having a cost per QALY gained of <£30 000. Combining emotional and cognitive benefits in the model's base case scenario the cost per QALY gained would be £5500 (McCabe, 2008). Other modelling work looking at universal use of social and emotional learning interventions for 11–16-year-old children, and drawing on a review of effectiveness evidence on its application to the prevention of bullying (Evers *et al.*, 2007), suggested that if the intervention reduces victimization by 15% then it would have an 92% of having a cost per QALY <£30 000 (Hummel *et al.*, 2009).

### Promoting mental health at the workplace

A number of reviews have looked at evaluations of the effectiveness of interventions delivered in the workplace to promote better mental health and well-being (Kuoppala *et al.*, 2008; Corbiere *et al.*, 2009; Martin *et al*., 2009a). Actions can be implemented at both an organizational level within the workplace and targeted at specific individuals. The former includes measures to promote awareness of the importance of mental health and well-being at work for managers, risk management for stress and poor mental health, for instance looking at job content, working conditions, terms of employment, social relations at work, modifications to physical working environment, flexible working hours, improved employer–employee communication and opportunities for career progression. Actions targeted at individuals can include modifying workloads, providing cognitive behavioural therapy, relaxation and meditation training, time management training, exercise programmes, journaling, biofeedback and goal setting.

Tables 3 and 4 summarize key findings on the economic case for investment in workplace mental health promotion from empirical and modelling-based studies. While the costs to business and to the economy in general of dealing poor mental health identified at work have been the focus of attention by policy makers in Europe and elsewhere in recent years (Dewa *et al.*, 2007; McDaid, 2007), less attention has been given to evaluating the economic costs and benefits of promoting positive mental health in the workplace. A recent review for NICE found no economic studies looking specifically at mental well-being at work had been published since 1990 (National Institute for Health and Clinical Excellence, 2009a).

**Table 3:** Economic analyses of primary studies evaluating interventions promoting mental health and well-being at work

Bibliographic information	Intervention (I) and comparator (C)	Target population and duration of economic analysis	Study design	Cost results	Mental health-related effectiveness results	Perspective/price year	Synthesis of costs and effectiveness data
(Loeppke *et al.*, 2008), USA	I: Health-risk assessment, lifestyle management, nurse telephone advice line and telephone nurse-led disease management	543 employees of company, matched with employees in other companies that were not enrolled in a health promotion programme	Observational study with matched controls	Costs of intervention are not reported	Overall improved health of workforce and significant reduction in overall levels of combined physical and mental health risk (*p*< 0.001)	Perspective not stated	Paper states that there are net savings after taking account of costs of intervention, but level of net savings not reported
	C: No intervention	3 years		Average decrease in 3.5 days per annum in absenteeism in the intervention group. No change in the control group. No significant difference in productivity at work	Majority of employees, where data available, maintained gains over 3 years	Price year not stated	
					Compared with control populations significant decrease in prevalence of depression from 17.9 to 10% (*p*< 0.01), but statistically significant increase for anxiety from 7.9 to 10.2% (*p*< 0.01)		
(McCraty *et al.*, 2009), USA	I: Power to change stress management and health-risk reduction programme. Includes emotion refocusing and restructuring techniques	75 correctional officers at a youth facility	Quasi-experimental study with waiting list controls	Cost of programme not reported	Intervention associated with improvements in scales measuring productivity (*p*< 0.01) motivation (*p*< 0.01), gratitude (*p*< 0.05), positive outlook (*p*< 0.05) and reductions in anger (*p*< 0.05) and fatigue (*p*< 0.05). In addition there was a significant increase in depression in the control group (*p*< 0.05)	Health system	43% of the intervention group had a sufficient reduction in number of risk factors to reduce projected health-care costs compared with just 26% of control group
	C: Waiting list	3 months	CCA	Projected average health-care cost per employee in the intervention group based on number of overall risk factors was reduced to $5377 from $6556. This compared with a reduction in from $6381 to $5995 in the control group		2004 USD	Intervention was associated with an average annual saving of $1179 per employee, compared with a reduction of $386 per employee in the control group (sample size too small for statistical significance on cost differences with controls)
(Mills *et al.*, 2007), England	I: A multi-component health promotion programme incorporating a health-risk appraisal questionnaire, access to a tailored health improvement web portal, wellness literature, and seminars and workshops focused upon identified wellness issues	1518 employees at the UK headquarters of a multi-national company	Before and after study	Annual cost of programme per company employee £70	Overall number of health-risk factors decreases significantly (by 0.48) in the intervention group	Company	Improved work performance and reduced absenteeism led to return of investment (ROI) of 6.19: 1
		12 months	CBA	Significant difference in absenteeism between control and intervention groups largely due to increase in absenteeism in the control group	Work performance also increased significantly by 0.61 points to 7.6 on work performance scale	GBP. Price year not stated	Net benefits of £621 per employee
					No significant changes in these outcomes in control groups		
(Munz *et al.*, 2001), USA	I: Comprehensive worksite stress management programme consisting of self-management training and an organizational level stressor reduction process	79 customer sales representatives in a telecommunications company	Non-randomized controlled trial; other work units were control groups	Costs of intervention not reported	Self-management training group had significantly less stress than control group on the perceived stress scale (2.63 versus 3.11) (*p*< 0.05). Significantly less likely to experience depression on the Centre for Epidemiological Studies-Depression Scale (CES-D) 11.60 versus 18.90 (*p*< 0.05). The training group also had significantly better levels of relaxation, positive energy and less tiredness than the control group using the positive and negative affect schedule (*p*< 0.05)	Not stated	No synthesis of costs and benefits. Significant improvement in emotional well-being in the intervention group compared with the control group;
	C: No intervention	3 months	COA	23% increase in sales revenue per order in the intervention group compared with 17% in the control group. 24% reduction in absenteeism in the intervention group compared with the control group	Individuals also had significantly greater sense of independence and job control in the intervention group (*p*< 0.05)		Benefits not reported in monetary terms, but at organizational level; 23% increase in sales revenue per order in the intervention group compared with 17% in the control group. Twenty four percent reduction in absenteeism in the intervention group compared with the control group.
(Naydeck *et al.*, 2008), USA	I: Comprehensive wellness programme including on-line sessions for nutrition, weight management, stress management, and smoking cessation; on-site classes in stress and weight management. Access to exercise facility and incentives to participate in walking programme	1892 employees who participated in company wellness programme. Matched controls from non-participants in company and non-participants in other companies	Observational study with matched controls	Total costs per employee per year were $138.74	No specific health benefits—mental or physical were reported—the study focused on reduction in overall health-care costs only of the wellness programme	Company as payer of health-care premiums for employees	Reduction in health-care costs over 4 years for the programme were $1 335 524, with net savings of $527 121 and a return on investment of $1.65
	C: No health promotion programme	4 years	COA, CBA			2005 USD	
(Ozminkowski *et al.*, 2002), USA	I: Multi-component Health and Wellness Programme including health profiles, risk management programmes and access to fitness centres, including financial incentives of up to $500 to participate in programmes	11 584 US-based employees of multi-national company	Before and after study making use of health claims data	Cost of programme not reported. Impact on health-care utilization reported. On average after 4 years overall reduction in health-care costs per worker of $224.66. This consisted of increase in cost of emergency department visits of $10.87; and decreases in costs of outpatient/doctor visits $45.17; mental health visits $70.69 and inpatient days of $119.67	Mental health (or other health-related outcomes) not reported. Instead changes in utilization of health-care services reported, including specific use of mental health service visits	Company (as health-care payer)	Investing in wellness programme associated with a large reduction in utilization of health-care services including mental health services over 4 years. On average savings per employee of $225 per year
	C: No intervention	60 months	COA	Impact on productivity not considered		2000 USD	Impacts on productivity not considered
(Rahe *et al.*, 2002), USA	I: Stress management programme focused on coping with stress through six group sessions and personal feedback	501 computer industry company and local city government employees	RCT	Cost of intervention $103 per employee	Stress, anxiety and coping levels improved significantly in all three groups after 12 months (*p*< 0.05), but there was no significant difference between groups with the exception of negative responses to stress for computer industry employees. Full intervention group computer industry employees had a significantly greater improvement in negative response, followed by partial intervention group and waiting list controls (*p*= 0.012)	Company perspective (as health-care payer)	No ratio reported, as no significant difference in stress, anxiety and coping
	C: Self-help groups with e-mail personal feedback (partial intervention) and waiting list control	12 months	CCA	Costs would be lower at $47.50 if delivered by in house medical professionals	There was a nearly significant difference in self-reported days of illness for the intervention group		But significant 34% reduction in health-care utilization by intervention participants compared with the control groups (*p*= 0.04)
							Concluded that this reduction in costs would more than cover the costs of delivering the intervention if delivered by in-house professionals
(Renaud *et al.*, 2008), Canada	I: Comprehensive health promotion programmes to provide employees with information and support for risk factor reduction, using a personalized approach and involving the organization's management as both programme participants and promoters. Programme includes modules on stress management, healthy eating and physical activity	270 company employees	Before and after study. No controls. COA	Cost of the intervention not reported Costs avoided not directly reported in monetary terms, but in terms of absenteeism and staff turnover	Significant reduction in stress levels away from work as reported using Global Health Profile Score over 3 years falling from 27 to 17% (*p*< 0.0001). There was also a reduction in feelings of depression with 54.8% of participants stating that they rarely felt depressed after 3 years compared with 38.5% at baseline (*p*< 0.0001). There was also a reduction in the number of people experiencing signs of stress (*p*< 0.0001)	Company perspective	No ratio. Significant reduction in high levels of stress, signs of stress and feelings of depression
	C: No control	3 years					Costs not directly reported staff absenteeism decreased by 28% and staff turnover by 54%
(van Rhenen *et al.*, 2007), Netherlands	I: Cognitive focused stress management programme	242 stressed and non-stressed employees of a telecommunications company	RCT	Costs not stated	No significant impact on sickness-related absenteeism between groups overall. Very marginally significant impact of cognitive interventions in delaying time to sickness	Company	Study authors commented costs not affected as overall no difference in impact on absenteeism
	C: Brief relaxation and physical exercise intervention	12 months	COA				

RCT, randomized controlled trial; CBA, cost–benefit analysis; CEA, cost-effectiveness analysis; CCA, cost-consequences analysis; CUA, cost–utility analysis; COA, cost-offset analysis.

**Table 4:** Economic modelling studies for interventions promoting mental health and well-being at work

Bibliographic information	Intervention (I) and comparator (C)	Sources of model parameters	Type of model and timeframe	Intervention cost	Perspective/price year	Economic results
		Study population	Economic analysis			
		Model timeframe				
(National Institute for Health and Clinical Excellence, 2009a). England	I: Comprehensive mental health promotion programme	Systematic review of literature for effectiveness data	Decision analytical modelling study	Cost of intervention not estimated, just costs averted	Company	Positive steps to improve the management of mental health in the workplace, including prevention and early identification of problems, could result in annual cost savings to company of 30%. In an organization with 1000 employees, this is equivalent to cost savings of £250 607 a year
	C: No intervention	Hypothetical company with 1000 employees	12 months		2009 GBPs	
			COA			

RCT, randomized controlled trial; CBA, cost–benefit analysis; CEA, cost-effectiveness analysis; CCA, cost-consequences analysis; CUA, cost–utility analysis; COA, cost-offset analysis.

In part this may be due to a lack of incentives for business to undertake such evaluations, as well as issues of commercial sensitivity. There have been few controlled trials of organizational workplace health promoting interventions, let alone interventions where mental health components can be identified and even fewer where information on the costs and consequences of the intervention are provided (Corbiere *et al.*, 2009). Moreover, many actions within the corporate world do not tend to be published in academic journals or books but rather in company literature. This makes studies more difficult to find and a full search of company literature was beyond the scope of our review. Most workplace health promotion evaluations related to mental health have focused on helping individuals already identified as having a mental health problem remain, enter or return to employment (Lo Sasso *et al.*, 2006; Wang *et al.*, 2006; Brouwers *et al.*, 2007; McDaid, 2007; Zechmeister *et al.*, 2008).

In fact, we were able to identify several economic analyses with some focus on mental health promotion (Table 3), largely from a US context where employers have had an not inconsiderable incentive to invest in workplace health promotion programmes, given that they typically have to pay health-care insurance premiums for their employees (Dewa *et al.*, 2007). At an organizational level, modelling work undertaken as part of the UK Foresight study on Mental Capital and Well-being suggests that substantial economic benefits that could arise from investment in stress and well-being audits, better integration of occupational and primary health-care systems and an extension in flexible working hours arrangements (Foresight Mental Capital and Wellbeing Project, 2008).

Modelling analysis of a comprehensive approach to promote mental well-being at work, quantifying some of the business case benefits of improved productivity and reduced absenteeism was also produced as part of guidance developed by NICE (Table 4). It suggested that productivity losses to employers as a result of undue stress and poor mental health could fall by 30%; for a 1000 employee company there would be a net reduction in costs in excess of €300 000 (National Institute for Health and Clinical Excellence, 2009b). Another analysis looking at the English NHS workforce reported potential economic gains from reducing absence levels down to levels seen in the private sector that would be equivalent to >15 000 additional staff being available every day to treat patients. This would amount to an annual cost saving to the English NHS of £500 million per annum (Boorman, 2009).

Most analyses have focused on actions targeted at individuals, such as stress management programmes, which are less complex to evaluate. There have been a number of economic assessments of general health promotion and wellness programmes (Pelletier, 1996, 2001, 2005, 2009; Chapman, 2005), but few have specifically mentioned mental well-being orientated components, and even when they do include these components they may not report mental health or even stress-specific outcomes. The Johnson and Johnson wellness programme, which includes stress management, has been associated with a reduction in health-care costs of $225 per employee per annum (Ozminkowski *et al.*, 2002), while a 4-year analysis of the Highmark company wellness programme, including stress management classes and online stress management advice, reported a return on every $1 invested of $1.65 when looking at the impact on health-care costs (Naydeck *et al.*, 2008). Neither analysis reported specific impacts on mental well-being or stress. Another study of an intervention to help cope with stress in the computer industry did not find any significant difference in stress levels, but it was associated with a significant reduction in overall reported illness and a one-third decrease in the use of health-care services which would more than cover the costs of the intervention (Rahe *et al.*, 2002).

One study that did report mental health outcomes looked at the economic case for investing in multi-component workplace-based health promotion programme (personalized health and well-being information and advice; health-risk appraisal questionnaire, access to a tailored health improvement web portal, wellness literature, and seminars and workshops focused on identified wellness issues). Using a pre-post test study design, participants were found to have significantly reduced health risks, including work-related stress and depression, reduced absenteeism and improved workplace performance. The cost of the intervention to the company was £70 per employee; there was a 6-fold return on investment due to a reduction in absenteeism and improvements in workplace productivity (Mills *et al.*, 2007).

The experience of employees in another health promotion scheme over 3 years was compared with matched controls. Overall levels of risk to health were significantly reduced, while there was also a significant reduction in the prevalence of depression, although rates of anxiety significantly increased. There were net cost savings from a health-care payer perspective, although the costs of participation in the health promotion programme were not reported (Loeppke *et al.*, 2008). In Canada, an uncontrolled evaluation of a comprehensive workplace health promotion programme, including information for stress management reported a significant reduction in stress levels, signs of stress and feelings of depression at the end of a 3-year study period. While costs of the programme were not reported, staff turnover and absenteeism decreased substantially (Renaud *et al.*, 2008). A small controlled study looking at a programme to prevent stress and poor health in correctional officers working in a youth detention facility in the USA, reported incremental cost savings of more than $1000 over 3 months, although the sample size was too small to be significant. However, the study did not monetize the value of reported productivity gains, while there were positive changes in outlook, attitudes, anger and fatigue (McCraty *et al.*, 2009).

Studies can also be identified where no impacts on absenteeism rates of stress management interventions were identified (van Rhenen *et al.*, 2007). In other cases analyses of a combination of organizational and individual stress management measures did report improvements in emotional well-being, as well as in productivity and reduced absenteeism, but no cost data were provided (Munz *et al.*, 2001). We also identified an ongoing cost–benefit analysis currently being conducted alongside a randomized controlled trial of a mental health promotion intervention to prevent depression targeted at managers in small and medium size companies involving cognitive behavioural therapy and delivered by DVD in Australia (Martin *et al*., 2009b).

### Investing in the mental health and well-being of older people

The final area we reviewed concerned the mental health and well-being of older people. Sixteen per cent of older people may have depression and related disorders; potentially the prevention of such depression, particularly among high-risk groups such as the bereaved, might help avoid significant costs to families, and health and social care systems (Smit *et al.*, 2006). Evaluations from a wider range of countries were identified, most notably from the Netherlands (Table 5). In addition to published studies discussed below, we also were able to identify some ongoing cost-effectiveness studies where protocols had already been published in open access journals (Joling *et al.*, 2008; Pot *et al.*, 2008).

**Table 5:** Economic analyses of interventions promoting mental health and well-being for older people

Bibliographic information	Intervention (I) and comparator (C)	Target population and duration of economic analysis	Study design	Cost results	Mental health-related effectiveness results	Perspective/price year	Synthesis of costs and effectiveness data
Baumgarten *et al*. (2002), Canada	I: Adult day-care programme. Included personalized programme of therapeutic and preventive activities, developed after in-depth evaluation of specific needs and abilities. Objectives to reduce psychosocial problems, keep ability to perform activities of daily living, maintain nutrition and exercise	280 patients older than 60 years of age, referred to any day centre	RCT	Mean cost of the services per client was CAD 2935 (±5536) in the intervention group and CAD 2138 (±4530) in the control group	Frequency of depression symptoms was measured using the Centre for Epidemiologic Studies Depression Scale (CES-D). There was a reduction in depression scores in both groups—16.9 to 16.5 in the intervention group, and 15.7 to 14.6 in the control group. No significant difference	Health, social and long-term care	No ratio reported as no significant difference in clinical outcomes or in costs. Intervention considered by authors as not shown to be cost-effective
	C: Usual care (not described)	3 months	CCA	These differences were not statistically significant	Anxiety scores on State-Trait Anxiety Scale went 39.7 to 39.2 in the intervention group, and 38.1 to 36.4 in the control group. No significant difference	1991 CAD	
					No significant change in functional status or in caregiver burden between the two groups		
(Bouman *et al*., 2008a, b), Netherlands	I: Eight home visits by home nurses with telephone follow-up.	330 community-dwelling people aged 70–84	RCT	Overall total cost per person, including the cost for the home visiting programme was €450 higher in the intervention group than in the control group. This difference was not statistically significant	Effectiveness analysis used a Self Rated Health Scale which looks at physical, mental and social functioning. No significant difference found in outcomes, but values not reported in paper	Health, social car and long-term care	No ratio reported as no significant difference in outcomes. On average intervention programme would have higher costs of €1525 but this was not statistically significant
	C: Usual care	24 months	CEA				Deemed to have only a 10% chance of being cost-effective
(Charlesworth *et al.*, 2008) and Wilson *et al.* (2009), England	I: Access to an employed befriending, facilitator and then offer of befriend in addition to usual care	236 carers of people with dementia (PwD). Mean age of carers was 68 years (range 36–91 years) and the mean age of PwD was older at 78 years	RCT	Total intervention cost at 15 months £122, 665; control group £120, 852. This difference was not significant	Depression and anxiety measured using Hospital Anxiety and Depression Scale (HADS). Positive affect measured using Positive and Negative Affect Schedule. Loneliness using Loneliness Scale	Societal, public purse, voluntary sector and household	Incremental cost per incremental QALY gained of £105 494. In sensitivity analysis, only a 42.2% probability of being below threshold of £30 000 per QALY gained.
	C: Usual care	15 months	CUA		Incremental Quality of Life Years (QALY) gained using EQ-5D over 15 months of 0.017 QALYs (0.946 versus 0.929). This was not significant		Not found to be effective nor cost-effective
(Cohen *et al.*, 2006), USA	I: Participation in choral singing group to promote mental and physical health	166 English language-speaking healthy community-dwelling people aged >65	RCT	Cost figures not stated but noted that significantly greater increase in doctor costs in the comparison group and lower increase in drug consumption in the intervention group	Philadelphia Geriatric Morale Scale; Geriatric Depression Scale Short Form; and engagement in social activities measured. Significantly lower decline in morale in the intervention group 14.15–14.08 versus 13.51–13.06 (*p*< 0.05). Significant reduction in loneliness 35.11–34.60 versus 38.26–37.02 (*p*< 0.1). No significant differences in depression, but significantly less decline in number of weekly activities in the intervention group 5.37–4.29 versus 4.88–2.58 (*p*< 0.01)	Health-care costs	No ratio but intervention dominant with better outcomes and lower costs than control group
	C: No action	12 months	Cost-offset analysis				
(Hay *et al.*, 2002), USA	I: Weekly group activity sessions by occupational therapists to promote positive changes in lifestyle. Topics included health behaviours, transportation, personal safety, social relationships, cultural awareness and finances.	163 ethnically diverse independent-healthy older people.	RCT	Programme costs $548 per person in OT group; $144 in social activity control group; $0 in passive control group.	Quality of life measured using the SF-36 and found to be statistically significantly in favour of OT group of 4.5% compared with combined controls (*p*< 0.001)—although actual QALY scores not reported in paper	Health and social care	Incremental cost per QALY gained with OT was $10 666 (95% CI: $6747–$25 430) over combined controls, $13 784 (95% CI: $7724–$57 879) over passive control group and $7820 (95% CI: $4993–$18025) over the social activity control
	C: (i) Social activity control group who undertook activity sessions including craft, films, outings, games, dances; (ii) no-treatment control group (*n*= 59)	9 months	CUA	Annual total costs (including health-care costs and healthcare costs to caregiver costs) were $4741in OT group, $3982 in social activity control group, $5388± passive control group and $4723 for combined control group). These differences were not statistically significant		1995 USD	
(Markle-Reid *et al.*, 2006), Canada	I: Nursing health promotion services bolster personal resources and environmental supports in order to reduce the level of vulnerability, enhance health and quality of life	288 people aged 75+ and newly referred to the Community Care Access Centre for personal support services	RCT	Costs figures not stated but noted no statistical difference in costs between groups	SF-36 used to measure physical and mental health. Center for Epidemiological Studies in Depression Scale—CES-D used to assess level of depression. There was a statistically significant average incremental improvement in SF-36 mental health score of 6.32 in the intervention group (10.8 versus 4.48)	Health and social care services	No ratio as costs not significantly different but better outcomes at same cost
	C: Usual home care services	6 months	CEA		Statistically significant reduction in mean depression symptom scores on CES-D score in intervention group of 2.72 (3.89 versus 1.17)		
(Munro *et al.*, 2004), England	I: Invitation to participate in free exercise classes every 2 weeks	20% least active older people in 12 primary care practices. 2283 in four practices were invited to exercise programme (of whom 590–26%—attended ≥1 session) and 4, 137 were controls	RCT	Mean costs €128 302/year, €125.78/session, €9.06/attender	Quality of life measured using the SF-36. Net significant QALY gains of 0.011 in the intervention group (*p*< 0.05)	Health-care payer perspective	Incremental cost per QALY gained of €17 174
	C: No invitation to participate	24 months	CUA	The incremental annual cost of the programme was €253 700 per 10 000 participants		2004. Euros, €s	
(Onrust *et al.*, 2008), Netherlands	I: Visiting service for older widow/ers bereaved for 6–9 months consisting of 10–12 home visits by a trained volunteer. Based on the Widow to Widow Programme	138 widows/78 widowers; 110 in the intervention group; 106 in the control group; Mean age of participants 68.8 (range 50–92)	RCT	Annual costs of intervention €553 per participant.	Quality of life measured using EQ-5D. Statistically significant improvement in QALYs gained in visiting service group (0.03; *p*= 0.025)	Health service costs, non-health patient costs (travelling, car parking etc); impact on ability to perform domestic tasks	Incremental cost per QALY gained €6827.
	Goal to bolster participant's personal resources through health assessment, managing risk factors and providing health education about lifestyles and disease management	24 months	CUA	Annual mean overall costs of €3220 versus €2389 between intervention and control groups. However, difference in change in costs over time between two groups, €210, not significant		Intervention costs included time of volunteers 2003. Euros, €s	Given a willingness to pay per QALY gained of €20 000; the intervention has a 70% of being cost-effective
	C: Brief brochure on depressive symptoms in addition to usual home care: case management, personal care, home support, nursing, occupational therapy, physiotherapy, social work and speech language therapy through community-based agencies						
(Pitkala *et al.*, 2009), Finland	I: Psychosocial group rehabilitation for older people experiencing loneliness. Aim to empower, promote peer support and social integration	235 community-dwelling older people (74 plus) experiencing loneliness	RCT	Costs associated with health-care utilization	Psychological well-being measured using a six-dimensional questionnaire. Psychological well-being score improved statistically significantly in the intervention groups +0.11 versus 0.01 (*p*< 0.05)	Health care	No ratio as intervention has better outcomes and lower health-care costs
	C: No action	12 months	COA	Significant net reduction in health-care costs of €943 per person per year (*p*< 0.05)		Euros, €s. Price year stated	
(Van't Veer-Tazelaar, 2010), Netherlands	I: Stepped care intervention to prevent depression: watchful waiting, bibliotherapy, problem-solving treatment and antidepressant medication	170 people; mean age 81.4; 70% women	RCT	Cost per patient of watchful waiting €26; bibliotherapy €259.25; problem-solving treatment €638.24; screening and referral to GP €59.36	Depression assessed MINI/DSM–IV diagnostic status of depressive and anxiety disorders. Probability of depression/anxiety-free year was 0.88 in intervention group versus 0.76 in the control group (*p*< 0.05).	Societal	Incremental cost per depression/anxiety-free year gained was €4367. 94% probability of being cost-effective if willing to spend €20 000 per depression/anxiety-free year gained
	C: Routine primary care	12 months	CEA	Mean total costs in the intervention group €2985; control group €2453		2007 Euros, €s	

RCT, randomized controlled trial; CBA, cost–benefit analysis; CEA, cost-effectiveness analysis; CCA, cost-consequences analysis; CUA, cost–utility analysis; COA, cost-offset analysis.

Several studies looked at different types of home visiting interventions to promote well-being and reduce the risk of depression, with mixed results. Neither a home visit programme by nurses in the Netherlands nor a programme to promote the befriending of older people in England was found to be effective or cost-effective (Bouman *et al*., 2008a, b; Charlesworth *et al.*, 2008; Wilson *et al.*, 2009). We did identify a cost–utility analysis from the Netherlands conducted alongside a randomized controlled trial comparing a home visiting service provided by trained volunteers with a brochure providing information on depression (Onrust *et al.*, 2008). It targeted older people who had been widowed for between 6 and 9 months and who were experiencing some degree of loneliness. Although improvements in quality of life were marginal, because of health service costs avoided the intervention had a 70% chance of being cost-effective, with a baseline cost per QALY gained of €6827 (2003 prices). In Canada a home nursing programme used to bolster personal resources and environmental supports of older people was also associated with a reduction in the risk of depression at no additional cost (Markle-Reid *et al.*, 2006). Recently a controlled trial of a stepped care approach for the prevention of depression in older people in the Netherlands was also found to be highly cost-effective at €4367 per depression/anxiety-free year gained (2007 prices) (Van't Veer-Tazelaar *et al.*, 2010).

Economic analyses also supported investment in some different types of group activities. Regular participation in exercise classes by older people was found to have some mental health benefits and be cost-effective from a health system perspective in England with a cost per QALY gained of €17 172 (2004 prices) (Munro *et al.*, 2004). Several studies also reported the beneficial effects to mental health of Tai Chi (MacFarlane *et al.*, 2005), but no formal cost-effectiveness analysis appears to have been undertaken. A study of 166 people randomized to participation in a choral singing group or no action was associated with a reduction in loneliness and lower health-care costs in the USA, albeit that the costs of the intervention were not estimated (Cohen *et al.*, 2006).Weekly group activity sessions led by occupational therapists in Canada significantly improved mental and physical health outcomes compared with participation in regular group social activities only. The incremental cost per QALY gained from a health and social care perspective was also considered to be cost-effective (Hay *et al.*, 2002). In Finland, a trial of psychosocial group therapy for older people identified to be lonely was also reported to be effective with a net mean reduction in health-care costs per participant of €943 (Pitkala *et al.*, 2009).

## DISCUSSION

Our review indicates that there is an economic evidence base in all of the areas examined by DataPrev for some interventions to promote mental health and well-being in some very specific contexts and settings. In addition, we were able to identify published protocols of additional economic studies now underway. However, much of the existing economic literature that is available was beyond the scope of this review as it focused on actions targeted at the prevention of further deterioration, as well as the alleviation of problems in people already identified as having clinical threshold levels of mental disorder. This is consistent with the findings of previous reviews (Zechmeister *et al.*, 2008).

One important limitation of our review was the restriction to English language only materials, although papers in other languages that had abstracts in English were included in the review. Certainly the overwhelming majority of material that we found came from English-speaking countries, but this is consistent with previous reviews of economic evaluations of public health interventions where no language restrictions were applied (McDaid and Needle, 2009).

We will have missed relevant studies concerning workplace interventions that have been published in diverse corporate literature with apparent positive returns on investment, but with insufficient information to be included in this review (Price Waterhouse Coopers, 2008). This includes case studies on the UK Health, Work and Wellbeing website looking at four large and small companies in the pharmaceutical, hotel and leisure, transport and manufacturing sectors. All report some positive impacts on absenteeism and/or staff retention rates. In the case of London Underground, for example, a return of 8:1 on investment in a stress management programme was reported (http://www.dwp.gov.uk/health-work-and-well-being/case-studies/).

Great caution must be exercised in drawing any firm conclusions on the economic case for investment, but the case for action in childhood or targeted at mothers appears strong. The economic consequences of poor mental health across different sectors and persisting into adulthood mean that effective health visiting and parenting programmes can have very favourable cost–benefit ratios; all economic analyses reported here from a societal perspective were cost-effective. Narrower perspectives adopted in some other child focused studies where evidence of effect was found, for instance from a health or education perspective alone, may undervalue the potential case for action.

Nine of the ten economic analysis set in the workplace reported favourable outcomes. Most of these studies looked solely at the impacts for employers, either in terms of paying for the health care of their employees or dealing with absenteeism and poor performance at work. No studies looking solely at the benefits of organizational level actions to promote well-being and mental health were found. Given that there is a literature on the effectiveness of some of these measures, there is scope for modelling work to look at the potential economic costs and benefits of these measures. Of the 10 studies looking at programmes for older people, 3 were found to have little chance of being cost-effective, but reasonable cost-effectiveness was reported for some group activities and home visiting activities.

In all areas we were able to identify published studies where no evidence of effect was found; these are also critical in helping to ensure resources are not used inappropriately. It is also the case that there has been little incentive to undertake formal economic evaluations of very low cost, but effective interventions, especially where costs are largely not borne by the public purse. One example of this are initiatives, often initially evaluated in low- and middle-income country contexts to promote skin-to-skin touch between mothers and their new born, where the principal cost is the time that the mother spends with her infant (Moore *et al.*, 2007; Maulik and Darmstadt, 2009).

Going forward our analysis of the methodological quality of studies suggests much room for improvement. While high-quality analyses were identified, most studies failed to separate presentation of data on resources used to deliver interventions from the costs of these resources. Few studies undertook more than a very cursory sensitivity analysis to account for uncertainty around estimates of effect and cost. There was little discussion of the distributional impacts of interventions, an issue that is of particular relevance in the context of public health and health promotion interventions, where engagement and uptake can be critical to effectiveness (McDaid and Sassi, 2010).

There is also a need for more common and consistent endpoints to improve comparability across different interventions and country settings. Reliance solely on topic-specific outcomes such the cost per unit improvement of maternal sensitivity or a reduction in loneliness mean that it is difficult to compare the case for different potential areas of intervention. One key challenge in economic analysis going forward is to develop measures that can adequately capture the benefits of improved mental well-being. The principal quality of life measure reported in studies here, the QALY, was designed to identify the benefits of the absence of illness rather than well-being. Work on other approaches to well-being is underway; but in the meantime making use of validated well-being instruments such as the Warwick-Edinburgh Mental Wellbeing Scale (Tennant *et al.*, 2007), alongside instruments used to value QALYs, such as the EQ-5D or SF-36, is merited. None of the cost–benefit analysis reported in this paper has elicited direct values for positive mental health: indeed the difficulty in putting a monetary value on well-being for cost–benefit analyses has been noted (Aos *et al.*, 2004). Another issue is that despite the links between poor physical and poor mental health, little economic analysis has focused on the economic case for preventing co-morbidity, for instance on the prevention of depression to promote cardiovascular health. This is another area that economists might explore further.

More use can also be made of economic modelling in the short term to help strengthen the evidence base for investing in mental health and well-being. Such an approach has recently been used to help inform policy making on the case for prevention of various mental health problems in both England and Australia (Knapp *et al.*, 2011; Mihalopoulos *et al.*, 2011). The DataPrev project has demonstrated that there is a substantial evidence base on effective interventions; most of these have not been subject to economic evaluation. Working with programme implementers to determine resource requirements, costs of delivery and any necessary local adaptations, economic models could be used to determine the likelihood that interventions are likely to be cost-effective in different contexts, and over different time periods. They can also be used to look at the case for investing in multi-level approaches to promotion and prevention, with some interventions targeted at the general population and others targeted solely at high-risk groups. Published examples of this approach include the Triple P programme for children (Mihalopoulos *et al.*, 2007; Foster *et al.*, 2008; Van't Veer-Tazelaar *et al.*, 2010) and stepped care for older people. Such models could also factor in key critical factors such as probability of uptake and continued engagement by different population groups.

## FUNDING

This work was supported by the European Commission Sixth Framework Research Programme. Contract SP5A-CT-2007–044145. Funding to pay the Open Access publication charges for this article was provided by the LSE Institutional Publication Fund.
